# Th2 Modulation of Transient Receptor Potential Channels: An Unmet Therapeutic Intervention for Atopic Dermatitis

**DOI:** 10.3389/fimmu.2021.696784

**Published:** 2021-06-30

**Authors:** Jianghui Meng, Yanqing Li, Michael J. M. Fischer, Martin Steinhoff, Weiwei Chen, Jiafu Wang

**Affiliations:** ^1^ School of Life Sciences, Henan University, Kaifeng, China; ^2^ National Institute for Cellular Biotechnology, Dublin City University, Dublin, Ireland; ^3^ Center for Physiology and Pharmacology, Medical University of Vienna, Vienna, Austria; ^4^ Department of Dermatology and Venereology, Hamad Medical Corporation, Doha, Qatar; ^5^ Translational Research Institute, Academic Health System, Hamad Medical Corporation, Doha, Qatar; ^6^ Dermatology Institute, Academic Health System, Hamad Medical Corporation, Doha, Qatar; ^7^ Department of Dermatology, Weill Cornell Medicine-Qatar, Doha, Qatar; ^8^ Qatar University, College of Medicine, Doha, Qatar; ^9^ Department of Dermatology, Weill Cornell Medicine, New York, NY, United States; ^10^ School of Biotechnology, Faculty of Science and Health, Dublin City University, Dublin, Ireland

**Keywords:** T helper-2, interleukin-13, interleukin-31, protease activated receptor 2, thymic stromal lymphopoietin, transient receptor potential channel, itch, pruritus

## Abstract

Atopic dermatitis (AD) is a multifaceted, chronic relapsing inflammatory skin disease that affects people of all ages. It is characterized by chronic eczema, constant pruritus, and severe discomfort. AD often progresses from mild annoyance to intractable pruritic inflammatory lesions associated with exacerbated skin sensitivity. The T helper-2 (Th2) response is mainly linked to the acute and subacute phase, whereas Th1 response has been associated in addition with the chronic phase. IL-17, IL-22, TSLP, and IL-31 also play a role in AD. Transient receptor potential (TRP) cation channels play a significant role in neuroinflammation, itch and pain, indicating neuroimmune circuits in AD. However, the Th2-driven cutaneous sensitization of TRP channels is underappreciated. Emerging findings suggest that critical Th2-related cytokines cause potentiation of TRP channels, thereby exaggerating inflammation and itch sensation. Evidence involves the following: (i) IL-13 enhances TRPV1 and TRPA1 transcription levels; (ii) IL-31 sensitizes TRPV1 *via* transcriptional and channel modulation, and indirectly modulates TRPV3 in keratinocytes; (iii) The Th2-cytokine TSLP increases TRPA1 synthesis in sensory neurons. These changes could be further enhanced by other Th2 cytokines, including IL-4, IL-25, and IL-33, which are inducers for IL-13, IL-31, or TSLP in skin. Taken together, this review highlights that Th2 cytokines potentiate TRP channels through diverse mechanisms under different inflammatory and pruritic conditions, and link this effect to distinct signaling cascades in AD. This review strengthens the notion that interrupting Th2-driven modulation of TRP channels will inhibit transition from acute to chronic AD, thereby aiding the development of effective therapeutics and treatment optimization.

## Introduction

Chronic-relapsing skin inflammation and intense itch are hallmarks in patients with atopic dermatitis (AD) (1–3). AD significantly impacts patient’s quality of life; however, the underlying mechanism is inadequately understood, particularly for patients in whom common treatments provide almost no relief.

In AD, pruritogens trigger somatosensory neurons to open transduction channels that depolarize the nerve terminal and promote action potential firing (4). In turn, these neurons release inflammatory mediators and signal to itch-specific neurons in the spinal cord. Subsequent pro-inflammatory response activates and sensitizes the transient receptor potential (TRP) channels, thereby worsens itching and inflammation.

During cutaneous neurogenic inflammation in chronic itch and AD, somatosensory afferents are activated by itch-producing compounds released by a variety of cells in the skin, including Th1/2 cells, Group 2 innate lymphoid cell (ILC2s), basophils, eosinophils, and mast cells (5). In addition, other cell types [e.g., keratinocytes and dendritic cells (DCs)] release inflammatory mediators in the periphery and further promote itch (6, 7). Altogether, these cascades shift AD from an acute phase to a systemic as well as neuropathic condition (4). Understanding the underlying molecular mechanisms and neuroimmune circuits helps to understand the transfer from acute to chronic condition, from non-lesional to lesional skin, and from chronic to recalcitrant itch.

The general consensus, to date, provides compelling evidence that inflammatory mediators can potentiate TRP channels. Particularly, Th1 cytokines involved in psoriasis or chronic phase of AD are known to potentiate TRP channels. However, to date, the role of Th2 cytokines in potentiation of these channels in AD is underappreciated. Recently emerged evidence suggests that several critical AD-associated Th2 cytokines potentiate TRP channel function in many ways. For instance, Th2 cytokines enhance TRP channel transcription and synthesis, rapidly modulate and sensitize channels (8). Th2 cytokines also regulate itch-selective peptide to modulate epidermal TRP channel function (9). Moreover, the TRP channel modulation is enhanced through an interplay between different Th2 or related cytokines (10). Thus, there is substantial evidence that the linkage of Th2 cytokines to sensory transducers like TRP channels substantially contributes to disease severity in AD. Hence, this review will help our understanding of chronic itch propagation, the basis how inflammation contributes to peripheral sensitization, and to develop new innovative strategies for the treatment of inflammation and itch in AD.

## Molecular Mechanism of Chronic Itch in AD: TH2 Cytokines and TRP Channels in Itch Sensation

AD itch results from dysregulation of neuro-immune circuits involving crosstalk between various receptors [TRP family members, Toll-like receptors, protease-activated receptor 2 (PAR2), IL-4R, IL-13R, IL-31RA, OSMR, Mas-related G proteins], itch peptides [substance P (SP), natriuretic peptide (BNP), and proteases], and pruritogenic cytokines [thymic stromal lymphopoietin (TSLP), IL-2, IL-4, IL-13, and IL-31] (11–13). Sensory nerves communicate with and can be activated by environmental factors including allergens, toxins, microbes, and pollution, thereby transmitting itch to the brain. The sensation of AD itch is mediated by the interplay between epidermal barrier dysfunction, upregulated immune cascades, and the activation of structures in the central nervous system. Endogenous or exogenous trigger factors of AD, such as protons, microbes, irritants or allergens can both directly or indirectly activate high-affinity receptors (e.g., TRP channels, Toll-like receptors, protease-activated receptors etc.) on peripheral sensory nerve endings (14, 15).

Th2 cells frequently reside in close anatomical vicinity to sensory skin nerve endings, whose somatosensory neuron cell bodies reside outside the spinal cord. Th2 cytokines including IL-4, IL-5 IL-13, IL-25, IL-31, TSLP, and periostin are the central mediators of human AD (16). These are also released by mast cells, basophils, eosinophils, ILC2s, keratinocytes, and are master regulators of chronic itch (17). IL-31 signaling in skin keratinocytes also dysregulates filaggrin expression and epidermal differentiation contributing to skin barrier dysfunction in AD, consequently leading to pruritus (18). TSLP induces DCs to release Th2-attracting chemokines (i.e. CCL17, CCL22), resulting in priming naïve T cells and subsequent release of Th2 cytokines (16). Cell expansion of Th2 central memory cells stimulated by TSLP-activated DCs can be promoted by type-2 immune responses, which is augmented by IL-25 (19). TSLP as a primary pruritogen pointing to an epidermal/immuno-neuronal communication pathway could feed into inflammation and itch in human AD. Mouse sensory neurons express TSLPR mostly in TRPA1^+^ neurons (10). In addition, TSLP triggers itch indirectly *via* stimulation of immune cells that release pruritogens such as IL-4 and IL-13, which in turn stimulate sensory neurons to induce pruritus (10). Interestingly, mechanical injury inflicted by tape stripping to the skin induced TSLP expression in murine keratinocytes, suggesting that the ‘vicious’ itch-scratch cycle observed in AD patients could in part be mediated and sustained by TSLP signaling (20). Accordingly, a recent study showed that TRPA1 knockout mice reveal a lower pruritus score by reducing the infiltration of mast cells, macrophages, as well as Th2 cytokine levels after challenge with 2,4-dinitrochlorobenzene (21).

Increasing evidence supports the evidence that many of Th cell-release cytokines are also produced by innate lymphoid cell subsets (e.g. ILC1, ILC2, ILC3). Group 2 ILC (ILC2s) are suggested to produce lots of Th2 cytokines, like Th2 cells, to contribute to the pathogenesis of type 2 dominated inflammation as seen in pathogenesis of AD (22). ILC2s express high levels of IL-5, IL-13, and the epidermal growth factor–like molecule amphiregulin (23), and receptors for IL-25, TSLP, and IL-33 (24), thus ILC2s also contribute to the Th2 cytokine-promoted TRP sensitization.

Activation of sensory nerve endings by Th2 pruritogenic cytokines leads to depolarization and electric “firing,” which in turn leads to the release of further neuro-mediators from central primary afferent nerve endings into the dorsal horn of the spinal cord (4, 25).

## T Helper Inflammatory Axis in AD and its Difference From Psoriasis

AD has been considered the paradigm of an allergic Th2-mediated disease, characterized by excessive IgE production, peripheral eosinophilia, mast cell activation, and induction of Th2 lymphocyte expressing IL-4, IL-10, IL-13, and so on, which promote cutaneous lesions from an acute phase (characterized by erythematous papules, intense itching, excoriation, and serous exudation) to a chronic phase (with lichenification) (1–3).

AD defects in the epidermal barrier result in a degree of inflammation, eczema, pruritus, dryness, and discomfort (26). Distinctive phenotypes, immunological, and genetic biomarkers, including cytokines, are essential for the classification of AD and chronic itch. Activated T helper cells in AD (mainly are Th2, less Th1, Th17, Th22) trigger the release of interleukins and other mediators of inflammation, which are essential markers in chronic AD. Th2-biased nature cytokines play important roles in the initiating stages of acute AD lesions and attract macrophages and eosinophils. A Th2 to Th1 switch promotes disease chronicity in the skin. Th1/Th2 dysbalance represents the immunological hallmark of AD. A mixed Th1 and Th2 cytokine pattern is implicated in chronic lesions (27). Th17 cytokines are also expressed in AD skin lesions but are less dominant than Th1 and Th2 cytokines, and they are rather linked to the acute than chronic lesions.

In contrast to AD, psoriasis is driven by Th1 and Th17 helper T cells. The expression of TRP channels (TRPV1, TRPA1, TRPV3) is elevated in both AD and psoriatic skin (9, 28–30). TRPV2 is upregulated in pruritic atopic skin, and TRPM8 is upregulated in pruritic psoriatic skin (28). The major difference between lesional skin samples from AD and psoriasis is that psoriasis plaques contain mainly cytokines secreted from Th1 and Th17 cells, for example, γ-interferon and IL-17, whereas AD skin samples contain relatively higher numbers of cytokines secreted from Th2 and Th22 cells (1, 2, 31). Since both AD and psoriasis are the common chronic itch diseases, thus, while this review talks about the updated mechanisms of Th2-mediated sensitization, we also discuss the current findings of Th1 and Th17 cytokines in potentiation of TRP channels to broaden our view on the difference in roles of Th1/Th17 and Th2 mediated TRP channel potentiation.

Systematic and critical analyses of the recent studies with focus on the mechanism of disease driven-modulation of hypersensitivity in chronic itch states in AD and comorbidities will aid the development of novel anti-itch therapeutics.

## Hypersensitization of TRP Channels

Considerable research has focused on TRP cation channels in sensory neurons and skin cells because of their pivotal roles in the transduction of itch signals. In the skin with AD, an increase of sensitivity and expression of certain members of the polymodal TRP ion channel superfamily is unveiled, especially, TRPV1, TRPA1, TRPV3, and TRPV4 (32–39). These channels play key roles in cell proliferation, inflammation, pain, and the propagation of itch signaling. With additional receptor overlaps, this generates several functional distinct populations, particular in sensory neurons, as revealed by RNA-seq (40), endowing afferent fibers with a complex array of polymodal capabilities.

In rat sensory neurons, the TRPV1-positive population is about double the size of the TRPA1-positive population, and the former largely engulfs the latter (41). TRPA1 expression is detected in dermal afferents and mast cells of mouse AD skin, and is essentially involved in chronic itch in mouse and human (36, 38, 42–44).

It has been reported that inflammatory agents, including nerve growth factor (NGF), bradykinin, insulin, and insulin-like growth factor 1, enhance sensory neuronal plasma membrane insertion of TRPV1 after undergoing phosphorylation by increased activity of certain kinases, such as protein kinase A or C, phosphatidylinositol 3-kinase, and/or sarcoma kinase (45–50).

Elevated expression of NGF and its receptors was observed in the basal layer of epidermis of involved AD skin, not in normal healthy volunteers, and AD uninvolved skin. This indicates their function in regulating immune response and inflammation, as well as in neuronally induced skin hyperplasia of AD (51). IL-31, which co-opts with TRPV1 and TRPA1 on sensory nerves, can also act as a neurotrophin by interacting with TRP channels (52).

Bradykinin is a classic endogenous algogen, which acts as a potent histamine-independent pruritogen in lesional AD skin and takes function in switching from pain to itch (53). Interestingly, in non-lesional skin of patients and in healthy volunteers, bradykinin evokes only weak itch and pain of similar intensities. However, in lesional skin, it induces intense itch which cannot be suppressed by the simultaneously increased pain, indicating an involvement of central sensitization (53).

In epidermal keratinocytes, TRPV3 is a dominant channel for chronic itch transmission that can be potentiated or sensitized by unsaturated fatty acids (54). Another pruriceptor-TRP in skin keratinocytes, TRPV4, functions differentially to the pathogenesis of chronic itch (55). Both TRPV3 and TRPV4 are also activated and potentiated by stimuli that are related to itch signaling, keratinocyte modulation, and inflammation (56, 57).

## AD-Related Cytokines in Modulation of TRP Channels

TRP channel modulation/potentiation has been observed in different stages associated by certain types of Th1, Th2, and Th17 cytokines, a mechanism underlying the progression and pathophysiological condition of AD (58). The Th1/Th2 dysbalance, indicative of a unique feature in AD, is of great importance in the manipulation of the TRP channel activation (31). AD begins with an acute phase that is signified by excessive Th2-dominated, but mixed with Th22 and Th17 cell activation. These T cells were increased in peripheral blood in patients with acute AD. In addition to the activated Th2 cells, increased ILC2s, basophils, eosinophils, and mast cells are also dominant sources during the acute stage of AD (58–62). These cells secrete large amount of Th2 cytokines, including IL-4, IL-5, IL-13, and IL-31. Epithelial cytokine IL-25, which can be released by dendritic cells, is also significantly up-regulated (63) and involved in Th2 inflammation. Moreover, IL-31 expression appears to be under control of AD-associated IL-4 and IL-33 (64).

When the acute phase of AD is switched to chronic phase subtype, Th1/Th17/Th22 inflammation response co-dominates. Th17 cells are characterized by the production of inflammatory cytokines, such as IL-17A, IL-17F, but also can secrete IL-22 and IL-26. Th22 cells produce cytokines, such as IL-22, IL-26, and IL-33 (65, 66).

A well-known Th1 pro-algesic cytokine that increases TRPV1 protein expression is TNF-α, which is released from mast cells, lymphocytes, macrophages as well as skin keratinocytes (67, 68). TNF-α enhances TRPV1 channel activity in rat dorsal root ganglionic (DRG) neurons and rat trigeminal ganglionic neurons (TGNs) (68–70). Additionally, TNF-α facilitates the plasma membrane insertion of TRPV1 and TRPA1 simultaneously in rat sensory neurons, thereby elevating skin hypersensitivity (7).

In terms of Th17-mediated TRP potentiation, an interesting cytokine is IL-6, which is produced by several cell types including antigen-presenting cells (APC), i.e., macrophages, dendritic, and B-cells. IL-6 upregulates TRPV1 in sensory neurons through activation of JAK/PI3K pathway (71). IL-6 level is increased in patients with AD and released in response to allergen challenge, thus being relevant for the acute-phase reaction of allergy (72, 73). IL-6 mediates activation of nuclear-factor of activated T-cells (NFkB), leading to the production of IL-4 by naive CD4(+) T cells and their differentiation into effector Th2 cells. Meanwhile, IL-6 inhibits Th1 differentiation through upregulating suppressor of cytokine signaling 1 (SOCS1) expression to interfere with the development of Th1 cells (74). Because of the link of IL-6 to Th2 and Th1 activation, intervening IL-6–mediated TRPV1 upregulation and hyperexcitability could be beneficial for the treatment of AD (75). In fact, antagonizing IL-6 signaling using its mAb tocilizumab decreased the clinical activity of severe AD in patients, highlighting its importance as the therapeutic target (75). However, the inhibition of IL-6 receptor by tocilizumab can improve AD in patients but associated with higher risk of bacterial infection (75).

Apart from IL-6, TRPV4 channel expression is stimulated by IL-17A, a Th17 cytokine involved in Th2-type immune responses in AD (76).

In contrast to the aforementioned Th1- and Th17-potentiation of TRP channels in AD and chronic itch, the Th2-driven potentiation of these TRP channels only recently attracted the attention of researchers. Accumulation of the released itch mediators and other inflammatory factors induced by Th2-cytokines in the early-onset, subclinical state can amplify the inflammatory response, to induce severe AD and itch (77). This could be attributed by sensitization and/or potentiation of TRPV1 and TRPA1 observed with heightened currents evoked by its agonists in sensory neurons (78). TRP channel expressing neurons activated by Th2 cytokines release inflammatory molecules and itch neuropeptides, and these cytokines and their downstream signaling molecules (i.e. substance P) also modulate the activity of TRP channels. These effects will exaggerate dermatitis and itch; therefore, the pathway from Th2 cytokines to TRP channels provides emerging targets for AD treatment.

Evidences reinforcing the information of Th2-driven modulation of TRP channels in progression of AD include IL-13–enhancing TRPV1 (79) and TRPA1 transcription (44), IL-31 transcriptionally regulating TRPV1 (80), and rapidly sensitizing TRPV1 channel activity (see below), and Pro-Th2 cytokine TSLP upregulating TRPA1 synthesis in sensory neurons (10). The modulation effect by the abovementioned Th2 cytokines could be further augmented by other IL-4, IL-25, and IL-33 cytokines, because these are the upstream regulators of IL-13 and IL-31 in AD skin. Moreover, IL-1β and TNF-α, which are known to potentiate TRP channels, can also induce TSLP release in human dendritic cells, human keratinocytes, and human skin explants (81, 82), resulting in compounded effects.

Taken together, Th2 inflammation plays critical roles in AD by directly activating pruriceptive sensory neurons and potentiating TRP channels to initiate pruritic inflammation and modulate the progression from acute to chronic stages. For example, IL-4Rα and IL-13Rα1 are expressed in sensory small diameter DRG neurons expressed in mice (40, 83–85), supporting the finding that IL-4 and IL-13 directly trigger sensory neurons (83, 86). IL-31 binds and activates a heterodimeric receptor composed of IL31RA and OSMRβ (87, 88), which is expressed by mouse sensory neurons (9). IL-31 activates TRPV1^+^/TRPA1^+^/IL31RA^+^ mouse sensory neurons to promote itch (8). TH2-derived IL-31 initiates STAT3-dependent proliferation, branching, and survival of small-diameter neurons in mice, underlying the basis for the increased sensory nerve fiber density in the skin of patients with AD (52). Mouse sensory neurons express TSLPR mostly in neurons that also co-express TRPA1 (10). TSLP can directly activate a subset of mouse TRPA1^+^ sensory neurons to elicit itch (10).

In order to fully understand the functions of cytokines in regulation TRP proteins in AD, the results of studies carried out to date are summarized in **Table 1**.

**Table 1:** AD-related cytokines in modulation of TRP channels.

Group	Cytokine	Pathological function in AD	Modulation of TRP channels	References
**Th1**	IL-1β	Serum levels increased in AD; induces TSLP and an AD-like phenotype in reconstructed healthy human epidermis; receptor expressions are associated with disease severity	Does not potentiate TRPV1; potentiates TRPA1 indirectly	(89, 90)
	TNF-α	Serum levels is increased in AD; induces TSLP expression in human keratinocyte; initiates the process of tethering, activation, and adhesion to the endothelium followed by extravasation of inflammatory cells	Increases TRPV1 expression; enhances TRPV1 activity; increases TRPV1 and TRPA1 membrane insertion in sensory neurons	(68–70, 91–93)
**Th17**	IL-6	Serum level is increased in AD; regulates immune response, inflammation, pathogen responses, bone metabolism, and hematopoiesis	Functionally upregulates TRPV1 expression in DRG neurons	(71, 75)
	IL-17	IL-17A decreases during the progression of AD from acute to chronic forms; triggers the production of IL-4 by Th2 cells; detected in acute AD lesions; number of peripheral blood IL-17^+^ CD4^+^ T cells correlated with disease severity; stimulates eosinophils to produce profibrotic cytokines	Neuronal IL-17 receptor upregulates TRPV4 but not TRPV1 receptors in DRG neurons	(76, 90, 94–97)
	TGF-β	Immunosuppressive; serum levels are increased during progression of acute AD to chronic forms; inhibits activity of Th1/2 cell types in human subjects; regulates TNF-α in mast cells and maturation of B cells; induces IL-31 expression from dermal dendritic cells to activate sensory neurons	Sensitizes TRPV1 through Cdk5 signaling in pulpal neurons and in DRG neurons	(80, 98, 99)
**Th2**	IL-4	The levels decrease during the progression of AD from acute to chronic forms; induces TSLP production in keratinocytes; activates sensory neurons; activates Th2 cells to release IL-4, 5, 6, 10, 13 to support allergic reaction; activates B cell to produce IgE	Indirectly potentiates TRPV1 and TRPA1 by inducing IL-13	(82, 83, 90, 100–104)
	IL-13	Involved in barrier dysfunction; induces TSLP production in keratinocytes; activates sensory neurons	Increases TRPV1 levels in lungs and bronchial epithelia of BALB/c mice; increases TRPA1 levels in mast cells.	(44, 79, 82, 90)
	IL-25	Epithelial cytokine; induces inflammation and skin barrier dysfunction; transgenic mice that overexpress IL-25 have elevated expression of IL-4, -5, and -13; dendritic IL-25 induces Th2 response and inhibits filaggrin synthesis, thereby affecting skin barrier function	May indirectly potentiate TRPV1 and TRPA1 though inducing release of IL-13	(90, 105, 106)
	IL-31	Itch inducer; elevated in AD lesions; enhances skin inflammation; leads to recruitment of T cells; induces IL-lβ, IL-6, CXCL1, CXCL8, CCL2, and CCL18 release from eosinophils	Increases TRPV1 expression in DRG neurons; potentiates proton-activated TRPV1 in DRG neurons.	(80, 107, 108)
	IL-33	Over-expressed in keratinocytes of patients with AD; stimulates Th2 lymphocytes, mast cells, and eosinophils to release IL-5, -13, and -31; promotes Th2-type immunity; reduces filaggrin and claudin-1 expression; reduces skin barrier function	Indirectly potentiate TRPV1 and TRPA1 though inducing release of IL-13 and IL-31	(90, 109, 110)
**Pro-Th2**	TSLP	Epithelial-derived; crucial for APC maturation; associated with autoimmune disorders; skews immune response toward Th2 phenotype; increases circulating levels of IL-4 and IL-13; activates subset of TRPV1^+^ and TRPA1^+^ sensory neurons	Upregulate TRPA1 synthesis in sensory neurons and promotes TRPA1 activity	(10)

## Mechanisms for TH2-Promoted TRP Channel Sensitization

Increased levels of IL-4, IL-5, IL-9, IL-13, IL-25, IL-31, IL-33, and the keratinocyte-derived factor TSLP, a master regulator of Th2-driven inflammation, have been identified in the skin of AD patients (100, 110, 111). These mediators are known to influence keratinocyte function and skin barrier integrity, which are prominent in atopic itch and hyper-pruritic condition (15).

In transgenic mice that overproduce Th2 cytokines, like IL-4, IL-5, IL-13, and IL-31, a positive correlation between the onset and progression of AD-like disease and the expression of these Th2 cytokines were observed (112). IL-4, IL-13, and TSLP directly activate sensory neurons to elicit or enhance itch sensation, indicative of a further hallmark of atopic skin (10, 83, 86). IL-31 induces pruritus by initiating the transduction (act as pruritogens) and sensitizes the TRP channels, thereby worsen the itch condition (8). IL-13 and IL-4 sensitize response of sensory neurons to many different pruritogens, including histamine (83). IL-4 or IL-13 induces the production of TSLP (82). In turn, TSLP triggers an inflammatory cascade, first, by activating myeloid DCs that provoke the proliferation of naïve CD4+ T cells, which then differentiate into Th2 cells that secrete inflammatory cytokines IL-4, IL-5, and IL-13 (113). The nature of Th2 cytokines being pruritogenic and the crosstalk between different Th2 cytokines further augments the potentiation of TRP channels, thereby contributing to the perpetuation of itch and neuro-inflammation.

### IL-13 Enhances TRPV1 and TRPA1 Expression in Itchy Skin

In the IL-13 transgenic mouse model of AD, intensive chronic itch is associated with enhanced expression of TRPA1 in dermal sensory nerve fibers, DRG neurons, and mast cells (83). TRPA1 expression is highly enhanced in epidermis from patients with lesional AD skin, in contrast to healthy subjects, might be attributed to IL-13 in induction of enhanced growth of dermal neuropeptide-secreting afferent nerve fibers (44). Moreover, IL-13 strongly induces the elevated expression of functional TRPA1 in mast cells (114). These findings highlight the complex interactions among dermal afferent nerves and mast cells in a Th2-dominated inflammatory environment (44). Nevertheless, it is not clear yet if rapid sensitization contributes to the IL-13 mediated itch condition and how this may differentially impacts sensory function.

Apart from TRPV1 and TRPA1, the levels of both IL-4 and IL-13, and epithelial-driven cytokines IL-25 and TSLP are also strongly correlated with expression of cold-activated TRPM8, a channel that is involved in non-neurogenic skin inflammation (115–117). As this is only observed in the sputum of patients with asthma so far, it remains unknown if IL-13 also modulates TRPM8 expression/function in AD. Nevertheless, this finding highlights that Th2 cytokines could also modulate TRPM8 expression levels.

### IL-31 Increases TRPV1 Expression and Rapidly Regulates Sensory Neuronal Activity

The transition from acute to chronic disease stages, the factors and mechanisms to shape chronic inflammatory activity and alter the responsiveness of sensory neurons, may not only depend on transcriptome global change over a long period but also on the modulation of sensitivity of TRP channels.

Overexpression of IL-31 is characterized by severe itch and chronic dermatitis (118). IL-31 receptor α (IL-31RA) is known to associate with Oncostatin M receptor (OSMR) to form the interleukin-31 receptor heterodimer complex to bind to IL-31 in mice (8). IL-31RA^+^/OSMR^+^ neurons are a small subset of TRPV1^+^-peptidergic sensory neurons (3, 8). IL-31 induces itch *via* the activation of TRPV1 and TRPA1 (8). IL-31 is an inducer of itching after cutaneous incision and is responsible for itch responses during wound healing (80), acts as a neurotrophin (52), and is involved in peripheral, as well as central pruritus (4, 88). In the skin wound, IL-31 increases expression of TRPV1 in 3 h after intradermal injection, and this potentiates calcium influx in DRG neurons, whereas expression of TRPA1 did not change significantly (80), highlighting IL-31 acts as a TRPV1 channel transcription modulator.

**Figure 1:**
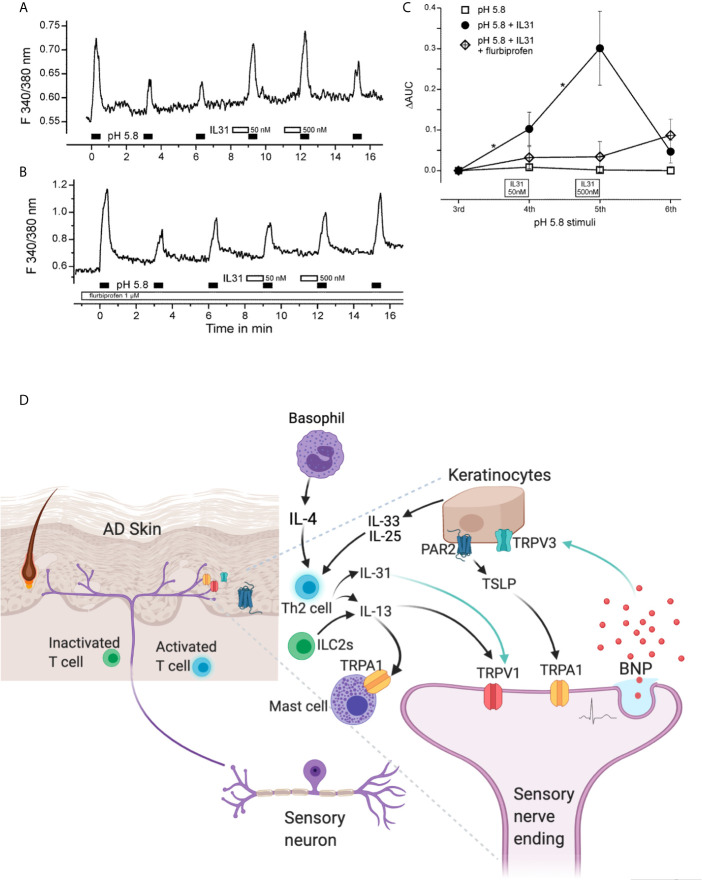
Th2-cytokine induced potentiation and sensitization of TRP channels in sensory neurons and skin. IL-31 potentiates TRPV1 in cultured murine DRG neurons. Calcium spikes on mDRG neurons excited with 200 ms exposure time at 1 Hz when continuously superfused with extracellular solution (145 mM NaCl, 1.25 mM CaCl2, 1 mM MgCl_2_, 5 mM KCl, 10 mM glucose, and 10 mM HEPES; adjusted to pH 7.3). IL-31 was diluted in this solution. Experimental procedures had been approved by the Medical University of Vienna Ethics Committee and local Authorities. **(A)** TRPV1 was repetitively stimulated by pH 5.8 solution. A 60 s pre-application of IL-31 dose-dependently potentiated acid-induced activation of TRPV1 based on AUC; **(B)** Abolishment of this sensitization in the presence of Cox1/2 blocker, flurbiprofen at 1 µM. **(C)** Quantitative analysis of panels **(A, B)**; changes in the AUC (ΔAUC) are presented relative to the third stimulation of **(A, B)**; n = 26, p = 0.009 *vs*. IL-31, ANOVA, post-hoc HSD; *P < 0.05. **(D)** IL-31 and IL-13, and PAR2-TSLP activation potentiate sensory neuronal TRPV1 and TRPA1 through fast and slow mechanism. In detail, disease-driven IL-31 upregulates TRPV1 synthesis and rapidly sensitizes TRPV1 channel function in sensory neurons. IL-13 enhances TRPA1 synthesis in mast cells. TSLP upregulates TRPA1 synthesis in sensory neurons. IL-31 induced BNP release from sensory neurons can increase the transcription level of TRPV3 in keratinocytes and elevate its activity. The Th2-cytokine mediated potentiation worsens pruritic and inflammatory conditions, resulting severe impairment of the skin barrier, increased susceptibility to infections, and elevated allergen sensitization in AD.

IL-31 activates TRPV1^+^/TRPA1^+^ sensory neurons to regulate pathogenesis of AD (8). In addition to the ability in driving itch-related neuropeptide release, it also acts as a sensitizer of TRP channel activity within seconds to minutes (6). In our finding, IL-31 modulated TRPV1 in DRG neurons in a fast mode. IL-31 potentiated proton (pH 5.8)-induced activation of TRPV1 in DRG neurons. Repeated application of pH 5.8 solution in DRG neurons for 30 s at intervals of 3 min resulted in increases in intracellular calcium with a stable amplitude after some initial tachyphylaxis (**Figure 1A**). After administration of IL-31, the pH 5.8 induced significant rise in calcium level compared to pH 5.8 alone. The analyzed data confirmed that IL-31 dose-dependently increased the area under the curve (AUC) of the spikes (**Figure 1B**). This effect requires activity of cyclooxygenase (COX) 1/2 because IL-31 effects were abrogated in the presence of the flurbiprofen (**Figure 1C**). Our finding supported IL-31 could re-sensitize TRPV1 channel in a fast mode. It suggests that during tissue damage and inflammation in AD, IL-31 can sensitize the pruriceptive neurons to respond to further stimuli, which itself is no longer sufficient to activate the neurons.

Taken together, Th2 mediators might have the ability to activate or sensitize nociceptive or pruriceptive nerve terminals to elicit itch and promote tenderness at the site of injury or skin lesion. Particularly, low pH (acidosis) is considered a hallmark of inflammatory responses and tissue acidosis implicated in AD. TRPV1-mediated proton sensing in tissues is physiologically relevant under normal conditions and in disease states of AD (119). Both local acidification as well as inflammation result in the lowering of the low PH threshold activating TRPV1 or bring it closer to activation.

Apart from low PH, aggravation of itch by warming temperatures is attributed by thermal sensor TRPV1 being sensitized by inflammatory factors in pathological condition in skin dermatitis, which possibly lowers the heat threshold to warmth sensation (120, 121) and increases the sensitivity to endogenous ligands in TRPV1 activation (122), to provoke itch in AD under environmental temperature changes.

### IL-31 Pathway Modulates Epidermal Keratinocyte TRPV3

Th2 immune responses could indirectly lead to the dermal remodeling and epidermal hyperplasia typical of chronic AD. An example can be seen from the IL-31 signaling-regulated TRPV3 hypersensitization.

Normal TRPV3 signaling is essential in maintaining homeostasis of the epidermal barrier. TRPV3 over-activation illustrates a critical cellular signaling cascade that directly influences normal cell proliferation, skin barrier formation, normal hair growth, release of immune mediators, and so on. This change of TRPV3 is seen in many cases of AD and similar skin diseases. The gain of function mutation of TRPV3 can alter or inhibit hair growth in the dermis, thus disturbing homeostasis of the skin barrier (123, 124). Loss of hair growth due to up-regulation of TRPV3 may further exaggerate pruritus in patients presenting with AD (9).

In AD, TRPV3 channel up-regulation in human keratinocytes in response to Th2 inflammation is thought to increase TSLP levels, and the upregulation might be attributed to NF-κB activation (30, 125). In human keratinocytes, PLCβ activation by G_q/11_ protein coupled receptor hugely enhances TRPV3 currents and M_1_ acetylcholine receptor stimulation increases the sensitivity of TRPV3 channel activation (126, 127). Such mechanism is also crucial for T-cell activation at sites of inflammation causing sensitization of TRPV3 channel.

Our findings have recently identified a neuro-immune modulatory cascade for amplification of TRPV3 synthesis and activity in mouse chronic itch (9). This involves IL-31 and itch-selective neuropeptide B-type natriuretic peptide (BNP) (9). First, IL-31 upregulates itch-related neuropeptide BNP synthesis and induces its release from mouse sensory neurons (6, 128) (**Figure 1D**). Second, BNP binds to its receptor NPR1 located on human keratinocytes and induces upregulation of TRPV3 transcriptional levels (9) (**Figure 1D**). Thirdly, BNP modifies TRPV3 activity and potentiates its mediated calcium influx (9) (**Figure 1D**). This cascade underlies a mechanism contributing to transition from acute itch to noxious stimulation, which then progresses to a prolonged itch and hyper-pruritic skin condition. We predicate that BNP increases TRPV3 levels on the cell surface probably through a vesicular transportation and membrane insertion (9). The IL-31-BNP-TRPV3 amplification cascade enhances Serpin E1 release from human keratinocytes. Serpin E1 is implicated in severe AD itch and has been identified as a new itch inducer in mice (9).

The link between IL-31 and TRPV3 through neuropeptide BNP might be a promising target for therapeutic development.

### The PAR2-TSLP Interaction Modulates TRP Channel Function

PAR2 has been extensively documented to promote Th2 inflammation and pruritus (129–131). In the skin of the PAR2 KO mice, the levels of the Th2 cell-secreted cytokines, IL-4, IL-5, and IL-13 were markedly reduced as compared to that from the wild type. Conversely, PAR2 activation induces a pro-Th2 cytokine TSLP, which is sufficient to trigger AD-like disease (10).

TSLP is an epithelial-derived cytokine, which is crucial for the maturation of antigen-presenting cells, and can be associated with various autoimmune disorders (132). TSLP may contribute to AD early in the course of the disease by causing itching, scratching, and breakdown of the skin barrier. It skews a T-helper immune response toward the Th2 phenotype and increases circulating levels of IL-4 and IL-13. Sensitivity to TSLP is significantly decreased in TRPA1-deficient neurons, but not TRPV1-deficient neurons (**Figure 1D**). TSLP activates a subset of TRPV1^+^/TRPA1^+^ sensory neurons and promotes TRPA1 activity *via* phospholipase C (PLC) signaling (10). As a support, inhibition of PLC not only reduces the prevalence of TSLP-sensitive neurons but also attenuates the TSLP-induced scratching (10).

PAR2 is a potent regulator for TRP channels as it indirectly modulates TRPA1 channel function (78), and also facilitates the activation and potentiation of TRPV1 and TRPV4 in mouse sensory neurons (133). When stimulated by PAR2 activator, co-regulation of TRPV1 and TRPV4 in a subset of mouse sensory neurons resulted in enhanced formation of functional complex to contribute to elevated histamine and chloroquine-induced itch (133). It is unclear whether facilitation by PAR2 on these channel proteins occurs at the plasma membrane or involves enhanced trafficking to the cell surface or how these channels are directed to the specific regions of the cell (133). Nevertheless, the PAR2 pathway by modulating TRP channel function represents a cutaneous feedback mechanism on sensory itch receptors to worsen AD severity (134).

### Hypersensitivity of TRP Channels Leads to Enhanced Neurogenic Inflammation, Increased Somatosensations, and Cutaneous Inflammation in AD

The hypersensitization of TRP family contributes to the increased somatosensations and persistent itch, neurogenic inflammation, and cutaneous skin inflammation (34). In AD, TRPA1 is increased in nerve fibers, keratinocytes, and tryptase positive mast cells from lesional skin of patients (44). TRPV1 is up-regulated in lesional skin (135) and increased sensitivity revealed (136). Activated TRP channels in mouse and rat sensory neurons release itch neuropeptides, including substance P and CGRP, both of which contribute to the characteristic flare and wheal that is concomitant with itch (85). Itch is also regulated by the VGLUT2-mediated transmission *via* the TRPV1^+^ neurons, through CGRP and gastrin-releasing peptide receptor (GRPR), the latter is a spinal itch receptor (137). The itch generation can be associated with different itch neurotransmitters combined and cooperate with each other to transmit or regulate itch sensation.

In sensory nerve, the identification of a subset of neurons as the dedicated itch-specific prurinergic fibers, named as MrgprA3^+^ neurons, is the first time to establish the existence of itch-specific nerves (138). In a mouse transgenic line that TRPV1 is only expressed in the MrgprA3^+^ neurons, the pain stimulator capsaicin no longer elicits nocifensive behavior, on the other hand, it elicits scratching behaviors (138). When the MrgprA3^+^ neurons are depleted, itch behaviors were reduced but thermal and mechanical allodynia was maintained. TRPA1 mediates histamine-independent, MrgprA3, and C11-dependent itch (139). The key player IL-31 enhances BNP release and synthesis and orchestrates cytokine and chemokine release from skin keratinocytes and DCs (6). IL-31–elicited itch behavior in mice is largely dependent on TRPV1 and TRPA1 channels as mice with either in TRPV1^−/−^ or TRPA1^−/−^ displayed significant reduction of scratching bouts (8).

In skin, TRP channels regulate cutaneous inflammation. Activation of cultured human primary keratinocytes by a TRPA1 agonist elicits pro-inflammatory cytokines, including IL-1 α and IL-1β (140). Noxious agents like ultraviolet light, thermal stimuli, low pH, endogenous bradykinin, NGF, lipids, and metabolites of arachidonic acid and ATP activate the TRP V1 receptor in the human keratinocyte resulting in the release of PGE 2, IL-8, and upregulate COX 2 together with other proinflammatory mediators (5, 141). TRPV2 is also found to be upregulated in the patient itchy atopic skin (28), and activation of TRPV2 channels causes mast cells (human cell line HMC-1) to degranulate (142). Keratinocytes isolated from AD patients display enhanced expression and heat sensitivity with hyperactive channel function of TRPV3. Agonists of TRPV3 increased IL-33 production, as well as TSLP, NGF, PGE2, in human keratinocytes and induced scratching behavior upon intradermal injection in mice (143). Activation of TRPV3 also triggers release of multiple factors, including PGE2, ATP, nitric oxide, and NGF, further contributing to the inflammation processes in dermatitis in skin level (144). Apart from this, TRPV4 is also selectively expressed by dermal macrophages and epidermal keratinocytes in mice, and critically and dynamically mediate chronic itch (55).

## Perspectives for Therapeutic Interventions Via Targeting Potentiation of TRP Channels

### Current Therapeutic Development Targeting Th Cytokines and TRP Channels for Treatment of AD and Itch

The treatment of chronic itch and AD is challenging because of the fact that it is a chronic relapsing disease, sometimes refractory to current treatments, adverse event from long-term topical and systemic therapies, and a lack of clear understanding about the exact mechanisms of pruritic mediation. Also, the involvement of underlying disease processes that carry their own complex etiologies further augments the problematic nature of treating chronic itch. Inhibition, desensitization, or downregulation (i.e., probably a consequence of anti-inflammatory therapies) of the TRP channels are possible therapeutic avenues, along with the identification of antagonists that can function to selectively inhibit signaling upstream of TRP channels, leading to promising antipruritic therapies (145).

Th2-cytokine modulation of TRP channels might be related to its intrinsic property as strong inflammatory progenitors. Th2 cytokines exhibit fast and slow modulatory effects for TRP channel potentiation through either potentiation of TRP channels *via* phosphorylation, thereby lowering the gating threshold, or increasing the plasma membrane translocation and presence of the channels at the membrane, or enhancement of channel transcription and protein synthesis, thereby increase the amount of channel trafficking and delivery to the plasma membrane.

Despite the importance of Th2 in potentiation of TRP channels, no therapeutics have been developed to interrupt the pro-inflammatory and pro-pruritic linkage of Th2-TRP. In fact, very limited therapeutics have been developed against TRP channels for itch and AD treatment (**Table 2**).

**Table 2:** Overview of Th-cytokine pathway-related and TRP channel-based anti-pruritic therapeutics investigated in both animal and clinical trials (Data are based on the ClinicalTrials.Gov 2021).

Mechanism of Action	Therapeutic	Route	Development Stage	Reference
IL-31RA antagonist	Nemolizumab	Systemic	Phase II	(146–154)
			NCT03100344 NCT04365387	
Anti-IL‐4Rα antibody (antagonist)	Dupilumab	Systemic	Phase II/III NCT04256759 NCT02277743 NCT02277769 NCT02260986 NCT03054428	(155, 156)
Anti-IL-17α antibody (antagonist)	Secukinumab	Systemic	Phase III/IV NCT01806597 NCT03440736 NCT02752776	(157–159)
IL-13 antagonist	Tralokinumab	Systemic	Phase III	(160, 161)
			NCT03363854	
IL-13 antagonist	Lebrikizumab	Systemic	Phase III	(162, 163)
			NCT04250337	
			NCT04146363	
TRPV1 antagonist	PAC-14028 (Asivatrep)	Topical	Phase III	(135, 164, 165)
			NCT02965118	
TRPV1 antagonist	SB-705498	Topical	Phase I NCT01673529	(166)
TRPV1 antagonist	AMG-9810	Topical	Animal study (mouse)	(167)
TRPV1 antagonist	SB366791	Systemic	Animal study (mouse)	(168)
TRPA1 antagonist	HC030031	Systemic	Animal study (mouse)	(38, 44, 168–170)
TRPA1 antagonist	A-967079	Systemic	Animal study (mouse)	(34, 38)
TRPV4 antagonist	HC-067047	Systemic	Animal Study (mouse)	(171–173)
TRPV4 antagonist	GSK2193874	Systemic	Animal Study (mouse)	(55, 174)
TRPV4 antagonist	GSK205	Systemic	Animal Study (mouse)	(175, 176)
TRPM8 agonist	Menthol solution	Topical	Animal Study (mouse)	(177)
TRPM8 agonist	ph5 Eucerin	Topical	Proof of Concept NCT00669708	(178)
NPRA and GRPR antagonists	A71915	Systemic	Animal (mouse) Study	(114, 179)
	RC-3095			
Anti-OSMR antibody (antagonist)	KPL-716	Systemic	Phase II NCT03858634 NCT03816891	(180, 181)
Anti-TSLP antibody (antagonist)	Tezepelumab	Systemic	Phase III NCT03809663(terminated)	(182, 183)

### Mechanism of TRP Family Antagonists as Therapeutics for AD Treatment

Because of the limited data available in clinical trials, the clinical efficacy and safety of TRP family antagonists in the treatment of AD remains to be explored. However, in the animal models of chronic and acute itch, as well as AD-like models, systemic and topical administration of TRP antagonists seem to have potential in improving symptoms of AD, recovering epidermal barrier function, and reducing itch-like behaviors.

TRPV1 is upregulated in lesional skin in murine AD models (135, 164). The blockade of activation of TRPV1 by PAC-14028 is confirmed in murine AD models induced by Dermatophagoides farina (Df)- and oxazolone (OXZ), whose AD-like symptoms have been improved, including serum IgE increase, mast cell degranulation, scratching behavior, and clinical severity of dermatitis (136). Another TRPV1 antagonist, AMD9810, was found to block excitation of sensory neurons and dramatically reduce scratching bouts in a mouse acute itch model induced by subcutaneous injection of immepip into the nape of the neck, but this is not observed for TRPA1 antagonist HC030031, suggesting that TRPV1 is implicated in histamine H4 receptor-mediated itch signaling (167). In AD, H4 receptor antagonists have shown both antipruritic and anti-inflammatory effects in murine models and in human clinical trials, implicating H4 receptors in AD (184, 185). TRPV1 agonist SB366791 and HC030031 alleviated PAR-4 agonist (AYPGKF-NH)-induced itch in mice, suggesting that this type of itch involves TRPV1/TRPA1-dependent mechanism (168). PAR4 is overexpressed in itchy AD skin, although the detailed function in AD remains to be defined (28). SB366791 may also block release of itch-related neuropeptide (SP) that is released by TRPV1/TRPA1 neurons at both peripheral and central levels. In IL-13–overexpressing transgenic mouse AD model, HC030031 administration markedly decreased IL-13–induced AD itch, and reduced TRPA1 expression in skin, but did not completely abrogate IL-13–induced itch responses, suggesting that TRPA1-independent mechanisms regulate the pathogenesis of IL-13–induced itch (44).

Skin dryness, excoriation, erythema, edema, cellular inflammatory responses, and histamine-independent pruritus are associated with OX-induced AD model. AD-like phenotypes were found to be attenuated by HC030031 in this model, but not by TRPV1 inhibitor, approving that TRPA1, but not TRPV1, is related closely to skin edema, keratinocyte hyperplasia, nerve growth, leukocyte infiltration, and histamine-independent scratching behavior in these mice (38). A similar observation was also obtained in mice exposed to the haptens, urushiol, and the contact allergen of poison ivy, which are the inducers for allergic contact dermatitis (ACD) (38).

A known neutrophil chemoattractant factor leukotriene B4 (LTB4)-induced acute itch (186), was found to be inhibited by SB366791 and TRPA1 antagonists TCS 5861528 and HC-030031 (169). LTB4 may initiate/amplify dermal inflammation, and abnormal T cell response (186, 187).. In addition, LTB4 is upregulated in AD lesions and is required for neutrophil recruitment to areas of injury or challenge, subsequent recruitment of CD4+ T cells, and Th2 inflammation (188).

Exposure to formaldehyde upregulates the activation of DRG neurons and exacerbates AD by inducing skin barrier dysfunction in AD patients (189). Repeated exposure of formaldehyde causes allergic contact dermatitis in both human (190) and animal models (191). In line with this, formalin-evoked acute itch in mice can be significantly reduced by HC030031 (172).

TRPV1 antagonist AMG517 aggravated the squaric acid dibutylester (SADBE)-induced skin inflammation, whereas it was not affected by administration of a selective TRPA1 channel blocker A967079, suggesting that TRPV1, but not TRPA1, plays a critical role in modulating ear edema in the SADBE-induced allergic contact dermatitis model (34). TRPV4 expression is elevated in skin samples from Human chronic idiopathic pruritus patients and TRPV4 function is required for generating mouse models of both allergic and nonallergic chronic itch (55). AEW-induced dry skin itch and SADBE-induced itch in mice both require TRPV4; therefore, the antagonist of TRPV4 alleviate scratching in these models (55). 5-hydroxytryptamine (5-HT) is an inflammatory and capable to induce itch in mice (not human), which is linked to TRPV4 function in epithelial and immune cells in skin, thus can be attenuated by TRPV4 antagonist HC067047 (173). Lysophosphatidylcholine (LPC)-induced scratching in mice was reduced by systemically TRPV4 inhibitors, GSK205 and HC067047, and the elevated LPC was previously observed in patients with AD (176).

Despite the fact many antagonists have been tested in different animal models, there is lack of inhibitor toward the potentiation of TRP family that can be used clinically. Moreover, even in different AD models, the effect of antagonists can be different, attributed by differential involvement of TRPV1 and TRPA1. For example, the phenotype of OX-induced murine AD requires TRPA1 (38), whereas SADBE-induced murine AD requires TRPV1 (34). The effectiveness of TRPA1 or TRPV1 in these models may not mimic/reflect the future findings in human AD that is characterized as a more complexed and heterogenous disease, in which the multiple cytokines may potentiates several TRP channels. Thus, an ubiquitous inhibitor that attenuate sensitization of TRP channel potentiation is needed. In this aspect, blockage the exocytotic delivery of TRP channels to the cell surface might be a promising stratagem.

### Future Therapeutic Against Potentiation of TRP Channels

**Figure 2:**
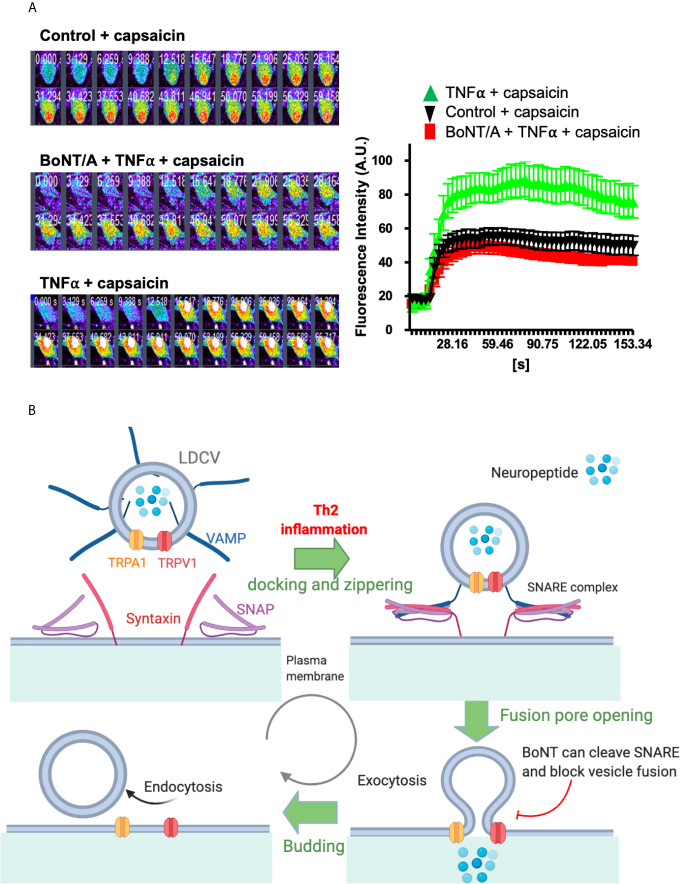
TNFα enhanced Ca^2+^ influx in cultured TGNs is blocked by truncation of SNAP-25. A model of SNARE mediated vesicle fusion and surface delivery of TRP channels mediated by inflammation. **(A)** Cultured rat TGNs, pre-treated with or without 100 nM botulinum neurotoxin A (BoNT/A), were incubated with TNFα for 24 h before measuring capsaicin-evoked Ca^2+^ influx with Fluo-4 AM, using confocal microscope imaging. Fluorescent readings (f) at each time point relative to the baseline (f_0_) are plotted. Note that capsaicin-elicited Ca^2+^-influx in TNFα-treated TGNs was normalized by BoNT/A pre-treatment. Data are the means ± S.E.M; >20 cells recorded from three independent culture preparations. Experimental procedures had been approved by the Dublin City University Ethics Committee and the Irish Authorities. **(B)** TRPV1 and TRPA1 mainly reside on the vesicle membrane of LDCVs that pack neuropeptides, including CGRP, SP or BNP. SNARE proteins (SNAP, VAMP, and syntaxin), and associated Munc-18 mediate the inflammation-stimulated vesicle trafficking and membrane fusion as well as the resultant membrane insertion of TRPV1 and TRPA1. Serotypes of BoNT that selectively cleave their respective SNAREs can block pain and itch-related neuropeptide release and membrane delivery of TRPV1 and TRPA1. This mechanism should aid the future designing of novel therapeutics for normalizing the increased surface appearance of itch transducing channels and the associated neuronal hyper-excitability upon inflammatory/pruritic stimulation. **(B)** Was modified from our previous paper (7).

Upregulation and activity-gated exocytotic vesicular insertion of channels contribute to increased TRP channel signaling in sensory neurons (192). Transportation of TRPV1 and TRPA1 occurred predominantly in large dense core vesicles (LDCVs) packing neuropeptides calcitonin gene-related peptide (CGRP) and substance P (SP) (7, 193). In neuronal subset expressing both channels, TRPA1 and TRPV1 are co-trafficked to the plasma membrane upon stimulation by an inflammatory cytokine. For example, TNFα and IL-1β enhance cell surface TRPV1/A1 *via* membrane fusion mediated by soluble N-ethylmaleimide-sensitive factor attachment protein receptor (SNAREs), which involve vesicle-associated membrane protein (VAMP1), synaptosomal-associated protein-25 kDa (SNAP-25), and possible syntaxin 1 (7). Interestingly, TRPV1 and TRPA1 channels are located in VAMP1- containing vesicles. Inflammatory cells released cytokine, such as TNFα, binds to its receptor on sensory neurons, resulting in activation of intracellular cascades, including MAPK, p38, and other translational factors to increase neuropeptide or itch mediator synthesis. These culminate in the enhancement of trafficking of TRPV1 or TRPA1-containing vesicles and release of neuropeptides plus probably other mediators. Enhancement by TNFα of Ca^2+^ influx through the upregulated surface-expressed TRPV1 and TRPA1 is normalized by truncation of SNAP-25 to disassemble SNARE complex (**Figure 2A**). It is reasonable to assume that equivalent processes apply in a sub-population of sensory neurons that contain one or other of these TRP channels. Under such conditions, the delivery of these channels to the neuronal surface *via* such processes is elevated; this cascade likely contributes to the genesis of severe itch.

Identification of peripheral components of AD has pushed forward the discovery of novel and effective therapeutics. Long-acting SNARE-cleaving botulinum neurotoxin (BoNT) is ideal therapeutic for breaking immune-nerve communication. There are seven serotypes of BoNTs (type/A to/G) present in nature. BoNT/A has the longest duration of action and sensory neurons are susceptible to BoNT/A. Our previous findings have demonstrated that BoNT/A can potently cleave SNAP-25 and inhibit depolarization-evoked pain-mediator release, e.g., substance P and CGRP (194). Most importantly, BoNT/A can also inhibit the cytokine-induced upregulation of TRPA1 and TRPV1 (7). It is likely the fast mode potentiation through Th2 cytokines mediated vesicle fusion contribute to the delivery of TRPV1 and TRPA1 channels to the plasma membrane, a mechanism that can be blocked by BoNTs (**Figure 2B**). In this content, Th2 mediator IL-31–elevated release of the important itch mediator BNP from sensory neurons, but not the basal release, requires SNAP-25 (6). Thus, it is reasonable to deduce that truncation of SNAP-25 by BoNT/A would also inhibit Th2 cytokine-evoked excessive BNP release from pruriceptive neurons and prevent the cutaneous itch sensitization by reducing the surface delivery of TRPA1 and TRPV1 (**Figure 2B**). Moreover, the BNP potentiation of TRPV3, which is partly mediated by vesicular membrane fusion with plasma-membrane of keratinocytes in skin, would also be prevented by BoNT protease/B or/D serotypes, which inactivate VAMPs. Thus, attenuation of TRPA1 and TRPV1 expression and their surface trafficking, plus inhibition of itch-related neuropeptide release from pruritic nerve would underlie the observed anti-pruritic activity of BoNTs in animal models of chronic itch and in patients with itch conditions (171, 174).

## Concluding Remarks

AD is a common chronic disease, which is associated with cutaneous inflammation and the unpleasant itch sensation. Distinctive phenotypes, as well as immunological and genetic biomarkers, are essential for the classification of AD and further highlight the need for personalized and targeted therapies for such skin diseases. The newly emerging antibody therapy is less cost-effective compared with traditional medicines, but the latter do not treat the underlying causes. Conversely, some forms of AD are resistant to the new antibody therapy, which is also associated with side effects. A prime outstanding question in the field involves the molecular mechanisms by which Th2 inflammation causes itch and inflammatory stimuli and potentiates pruriceptive TRP sensors. The newly emerging evidence on Th2-TRP channel linkage provide promising scope for therapeutic development. Interrupting neuronal type-2 cytokine signaling on TRP channel sensitization could ameliorate pathologic AD and an effective strategy to target chronic itch.

## Author Contributions

Supervision: JM and JW. Conceptualization: JM, JW, and MS. Data curation: JM, YL, MF, WC, and JW. Funding acquisition: YL, JM, WC, JW, and MS. Writing—original draft preparation: All authors. Writing—review and editing: All authors. All authors contributed to the article and approved the submitted version.

## Funding

This work is supported by a funding from Henan University and a funding from the Science Foundation Ireland (15/SIRG/3508T) and a Type-2 Innovation Grant from Sanofi Genzyme (to JM), and National Priorities Research Program (NPRP11S-0117-180326) of Qatar National Research Fund, Member of Qatar Foundation, Internal Research Grand Competition (IRGC-04-SI-17-151) of the MRC Fund, Hamad Medical Corporation, Qatar (to MS).

## Conflict of Interest

Author MS was employed by company Hamad Medical Corporation.

The remaining authors declare that the research was conducted in the absence of any commercial or financial relationships that could be construed as a potential conflict of interest.

## Abbreviations

AD, atopic dermatitis; BoNT, botulinum neurotoxin; BNP, B-type natriuretic peptide; CGRP, calcitonin gene-related peptide; CNI, cutaneous neurogenic inflammation; COX, cyclooxygenase; DC, dendritic cells; DRG, dorsal root ganglia; FDA, Food and Drug Administration; GRPR, gastrin-releasing peptide receptor; ILC, innate lymphoid cell; LDCVs, large dense core vesicles; LPC, lysophosphatidylcholine; LTB4, leukotriene B4; NGF, nerve growth factor; OSMR, oncostatin M receptor; PAR2, protease activated receptor 2; PGE2, prostaglandin E2; PLC, phospholipase C; SADBE, aggravated the squaric acid dibutylester; SNAP-25, synaptosomal associated protein-25 kDa; SNAREs, soluble N-ethylmaleimide-sensitive factor attachment protein receptor; Th, T helper cells; TRP, transient receptor potential cation channels; TRPV1, transient receptor potential cation channel, subfamily V, member 1; TSLP, thymic stromal lymphopoietin; TSLPR, thymic stromal lymphopoietin receptor; VAMP1, vesicle-associated membrane protein.
